# Intracellular *Streptococcus pyogenes* in Human Macrophages Display an Altered Gene Expression Profile

**DOI:** 10.1371/journal.pone.0035218

**Published:** 2012-04-12

**Authors:** Erika Hertzén, Linda Johansson, Rita Kansal, Alexander Hecht, Samira Dahesh, Marton Janos, Victor Nizet, Malak Kotb, Anna Norrby-Teglund

**Affiliations:** 1 Center for Infectious Medicine, Karolinska Institutet, Stockholm, Sweden; 2 The Cincinnati A Medical Center and the Department of Molecular Genetics, Biochemistry and Microbiology, University of Cincinnati, Cincinnati, Ohio, United States of America; 3 Department of Pediatrics and Skaggs School of Pharmacy and Pharmaceutical Sciences, University of California San Diego, La Jolla, California, United States of America; Aarhus University, Denmark

## Abstract

*Streptococcus pyogenes* is an important human pathogen, which has recently gained recognition as an intracellular microorganism during the course of severe invasive infections such as necrotizing fasciitis. Although the surface anchored M protein has been identified as a pivotal factor affecting phagosomal maturation and *S. pyogenes* survival within macrophages, the overall transcriptional profile required for the pathogen to adapt and persist intracellularly is as of yet unknown. To address this, the gene expression profile of *S. pyogenes* within human macrophages was determined and compared to that of extracellular bacteria using customized microarrays and real-time qRT-PCR. In order to model the early phase of infection involving adaptation to the intracellular compartment, samples were collected 2h post-infection. Microarray analysis revealed that the expression of 145 streptococcal genes was significantly altered in the intracellular environment. The majority of differentially regulated genes were associated with metabolic and energy-dependent processes. Key up-regulated genes in early phase intracellular bacteria were *ihk* and *irr*, encoding a two-component gene regulatory system (TCS). Comparison of gene expression of selected genes at 2h and 6h post-infection revealed a dramatic shift in response regulators over time with a down-regulation of *ihk/irr* genes concurring with an up-regulation of the *cov*R/S TCS. In re-infection assays, intracellular bacteria from the 6h time point exhibited significantly greater survival within macrophages than did bacteria collected at the 2h time point. An isogenic *S. pyogenes* mutant deficient in *ihk/irr* displayed significantly reduced bacterial counts when compared to wild-type bacteria following infection of macrophages. The findings illustrate how gene expression of *S. pyogenes* during the intracellular life cycle is fine-tuned by temporal expression of specific two-component systems.

## Introduction

*S. pyogenes* is an important human pathogen causing an estimated 500,000 deaths yearly [1]. The bacterium is associated with a wide disease spectrum ranging from mild infections of the skin and mucosa such as impetigo and tonsillitis [2] to severe invasive infections associated with high mortality, such as toxic shock syndrome and necrotizing faciitis [3], [4]. Although *S. pyogenes* is often found extracellulary in the host, we and others have demonstrated that the pathogen can reside intracellularly and circumvent bacterial killing in both human macrophages [5], [6] and neutrophils [7], [8]. In macrophages, *S. pyogenes* resides within phagocytic vacuoles that not only serve as safe-havens but also niches for replication [6]; thereby contributing to bacterial persistence during invasive infection. In these phagocytic cells, the bacterial surface anchored M protein was recognized as an important factor contributing to *S. pyogenes* intracellular survival [6], [7].

During the infection process, it is of importance for bacteria to efficiently adapt to the changing environments within the host. Pathogenic bacteria have been shown to employ various mechanisms to fine-tune expression of virulence factors in response to growth conditions and stresses encountered in different niches and stages of infection [9]. Normally, a distinct set of virulence factors are expressed during each stage of infection, many of which are regulated by TCSs. The TCSs generally consist of a sensory and a regulatory component where the sensor component is typically a transmembrane histidine kinase that recognizes one or more environmental signals, such as changes in ion concentration, osmotic pressure or pH [10]. The majority of response regulator are cytoplasmic transcription factors that may either repress or activate transcription of target genes [9], [10]. In *S. pyogenes*, 13 different TCSs have been mapped, out of which 4 have been studied in more detail: CovR/S, FasBCAX, SptR/S and Ihk/Irr. The best characterized is the control of virulence (CovR/S) system, also known as CsrR/S [11], [12]. CovR/S affects around 15% of the bacterial genome and acts primarily by repressing transcription of virulence factors such as the hyaluronic acid capsule, streptokinase, streptolysins O and S, and cysteine protease Mac/IdeS [12]–[14]. Recently, CovR/S has received considerable attention due to the emergence of specific hyper-virulent streptococcal strains associated with *in vivo* selection of mutations in *covS* [14]–[19]. FasBCAX regulates fibronectin/fibrinogen binding, hemolytic activity and streptokinase transcription [20], SptR/S has been shown to be important for the persistence of *S. pyogenes* in human saliva [21] and the Ihk/Irr was recently reported to be important for *S. pyogenes* resistance to neutrophil killing [22].

Once inside the cell, bacterial survival and replication depend on significant metabolic adaptation, so that available nutrients can be efficiently utilized. As human bacterial pathogens are heterotrophs, there is a constant need for carbon inside the cell to serve as energy sources and as substrates for the production of the different macromolecules, such as protein and cell envelope components utilized by the pathogen. The major carbon and nitrogen substrates are normally transported by specialized pathways and uptake systems, such as ATP-binding cassette (ABC) transporters and phosphotransferase systems (reviewed in [23]). In *S. pyogenes* (strain SF370), 36 ABC transporters have been identified, and their functions suggested to be associated with systems regulating transport of iron, phosphate, inorganic ions, sugars, dipeptides/oligopeptides and amino acids [24].

We have previously shown that *S. pyogenes* can survive inside human macrophages through M protein dependent inhibition of phagosomal maturation [6]. In the present study, we sought to identify bacterial genes involved during early stages of intracellular persistence in macrophages, i.e. the primary host cell harboring *S. pyogenes* at the tissue site of infection [5]. Using microarrays we established the gene expression profile of intracellular *S. pyogenes* in human macrophages. Our results revealed a differential gene expression profile with 145 altered genes in intracellular as compared to extracellular bacteria. Notably, we observed significant up-regulation of the *ihk/irr* TCS in intracellular *S. pyogenes* at the early phase of infection. Our results suggest a multifaceted bacterial adaptation to the intracellular environment in macrophages.

## Results

### Differential Gene Expression Patterns between Intracellular and Extracellular Bacteria

**Figure 1:**
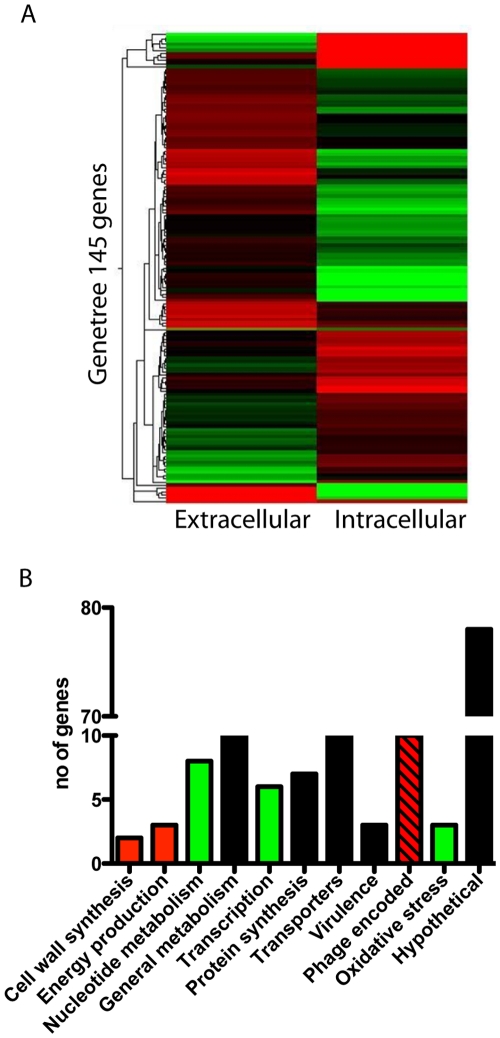
Differential gene expression profile in intracellular versus extracellular bacteria. **A**) Heat map of genes (n = 145) that displayed significantly (*P* : < 0.05) altered expression in intracellular versus extracellular *S. pyogenes* at 2h pi. RNA from 3 biological replicates were hybridized to microarrays and analyzed as described in the material and method section. Red color indicates up-regulation of genes; green down-regulation and black colored genes are not active as compared to house-keeping genes in the respective condition. **B**) Bar graph displaying differently expressed genes according to functional categories. Red bars indicate ≥75% of the genes are up-regulated, green bars indicate that ≥75% of the genes are down-regulated and black bars indicate that there is no consistent trend within the category.

**Figure 2:**
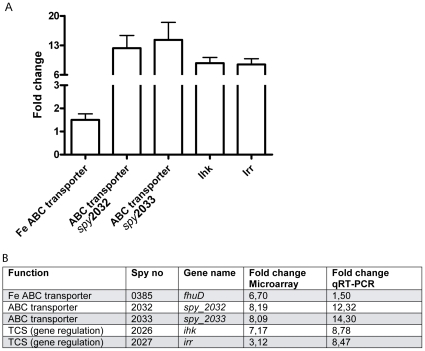
Two component system and transporter up-regulation confirmed with qRT-PCR. **A**) Expression of *ihk, irr,* and ABC transporters in intracellular 2h pi and extracellular bacteria determined by qRT-PCR analysis. Normalized mean value of 4 biological replicates shown as fold change in intra- versus extracellular bacteria. **B**) Expression of selected genes at 2h pi determined by qRT-PCR or microarray. RNA from 3 - 4 biological replicates per method was analyzed. The normalized mean value for each gene is found in the table.

The infectious cycle of bacteria within host cells involves several distinct phases, where each phase is likely to be associated with specific gene expression profiles [13]. Here we aim to model the early adaptive phase that is required to support intracellular survival. For this purpose, bacterial transcriptome profiles were determined 2h post-infection (pi) of primary human monocyte-derived macrophages. Only intracellular bacteria were taken into account, as extracellular or adherent bacteria were killed off by antibiotics. All infections were done with the clinical *S. pyogenes* 5448 isolate of the M1T1 serotype, previously well-characterized with respect to intracellular trafficking and survival in macrophages [6]. Extracellular control bacteria were cultured without cells under identical growth conditions. Bacterial RNA was reversely transcribed into cDNA and hybridized to customized microarrays of printed oligonucleotides [13], [14]. We observed stark differences in gene expression profiles of intracellular bacteria when compared to extracellular bacteria with 145 genes having a significantly altered expression (*P* < 0.05) (Fig. 1A). The majority of the up-regulated genes in intracellular bacteria were involved in cell wall synthesis and energy production (Fig. 1B). Phage-encoded genes is another category in which many genes were found to be up-regulated, some as high as 50-fold. The genes were associated with different prophages, including Ф70.1, Ф370.2 and Ф370.3. The two most strongly up-regulated were Spy0676 and Spy0698 (20 and 50-fold, respectively), both associated with Ф370.1. These genes, as well as most of the other deregulated phage-associated genes, encoded for hypothetical proteins with unknown function. The phage-encoded category also included a transcriptional repressor, which was down-regulated 4-fold. In contrast, genes associated with nucleotide metabolism, transcription and oxidative stress were in general down-regulated in intracellular *S. pyogenes* when compared to extracellular bacteria (Fig. 1B). Other functional categories such general metabolism, protein synthesis and transporters varied greatly and no trend of up- or down-regulation could be seen observed (Fig 1B). However, some genes were clearly differentially expressed. These include the ABC transporters (Spy2032/2033 and Spy0385), which are of importance for efficient metabolism due to their involvement in import and export of nutrients as well as in transport of ions. Our microarray data revealed an 8-fold up-regulated activity of an ABC transporter (Spy 2032/2033) and of the iron ABC transporter (Spy0385) (Fig. 2A). Another interesting finding was that the TCS *ihk/irr* genes (Spy2026/2027) were respectively 7- and 3-fold up-regulated in intracellular *S. pyogenes* (Fig. 2A). To validate these data, new infection assays were performed from which RNA was extracted and analyzed for selected genes using TaqMan qRT-PCR. In agreement with the previous result, *ihk/irr* genes and the ABC transporters all showed marked up-regulated expression ranging between 8–14-fold in intracellular when compared to extracellular bacteria (Fig. 2B).

The Ihk/Irr TCS has previously been reported to influence expression of 351 genes, as identified by microarray analyses of wildtype and *irr*-deficient *S. pyogenes* mutant strains [25]. Here we find that 22 of the 145 differentially expressed genes in intracellular *S. pyogenes* 2h pi are among the Ihk/Irr regulated genes (Table 1). Several of these genes did not have the same regulatory pattern (up– or down-regulation), which is not surprising considering the large variation seen between early and late exponential phase, as reported by Voyich et al [25]. However, 9 genes did show the same regulatory pattern, most being identified during the early exponential phase and involved in biosynthesis or metabolism (Table 1).

**Table 1:** Comparison of previously identified Ihk/Irr regulated genes [25] with differentially expressed genes of intracellular wildtype bacteria at 2h pi.

Spy no	Encoded protein	This study, fold change^a^	Voyich et al [25], fold change & growth phase^b^
*Nucleotide biosynthesis (5)*			
0901	Orotate phosphoriosyltransferase	+ 11,11	−1,7 EE
2105	Anaerobic ribonucleoside triphosphate reductase activating protein	−5,71	+1,6 LE
**0808**	**Metyl transferase**	**+2.00**	**+1,9 EE**
**1984**	**Nrdl protein, ribonucleotide reductase**	**−4,49**	**−1,5 LE**
**0024**	**Phosphoribosylaminoimidazole succicarboxamide synthase**	**−5,14**	**−2,1 EE**
*General metabolism (4)*			
0341	GTP-binding protein	−3,40	+1,5 LE
**1811**	**Fructokinase 4**	**−3,48**	**−1,6 EE**
**1754**	**3-oxoacyl-synthase III**	**−1,92**	**−1,8 EE**
1849	Formate acteyltransferase	−3,07	+1,5 EE
*Protein synthesis (2)*			
**0069**	**Ribosomal protein S5**	**+4,46**	**+1,9 EE**
0067	Ribosoaml protein L18	+4,80	+1,6 EE + 1,6 LE
*Transporters (3)*			
**1828**	**Putative transporter**	**−9,27**	**−1,7 EE**
0287	ABC transporter	−2,40	+1,6 LE
0831	Uracil permease	+1,52	−1,7 EE
*Cell wall synthesis (1)*			
0390	Low temperature requirement B protein	−2,60	+1,8 LE
*Hypothetical protein (7)*			
1146		+1,86	+1,8 LE
**0407**		**+1,25**	**+1,9 EE + 1,6 LE**
1455	Prophage encoded	–2,65	+1,5 LE
1686		–2,51	+1,8 EE + 2,2 LE
1516	Integral membrane	–2,08	+ 2,2 LE
**M18-1508**		**+61,81**	**+1,6 LE**
0914		–6,63	+1,7 EE + 1,8 LE

a Microarray array data.

b Gene expression in wildtype and *irr*-deficient *S. pyogenes* strains was determined at both early and late exponential growth phase, indicated by EE and LE resp.

Genes that show the same regulatory pattern in this study and in Voyich et al. [25] are indicated in bold.

### Fine-tuned Gene Regulation through Temporal Expression of TCSs During the Infectious Cycle

**Figure 3:**
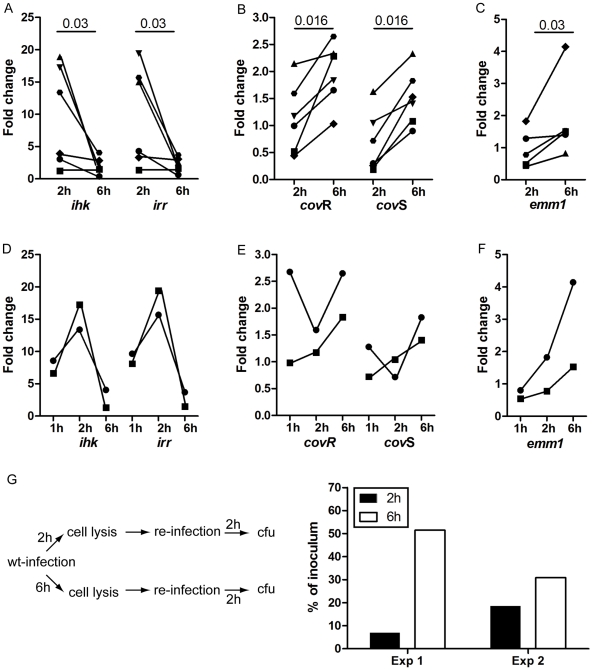
Shift in gene regulatory systems as the infection proceeds influence infectivity. RNA from intracellular and extracellular *S. pyogenes* was isolated at 2h and 6h pi. **A**) shows qRT-PCR data from *ihk/irr,* **B**) *covR/S,* and **C**) *emm1.* 6 biological replicates were analyzed with each sample in triplicate per gene. Statistical significant differences were determined by Wilcoxon signed rank test, and *p* values are indicated in the figure. Additional experiments included one additional time point at 1h pi and was analyzed for two donors showing *ihk/irr* **D**), *covR/S* **E**) and *emm1* **F**). Monocyte-derived macrophages were infected for 2h and 6h, respectively, after which the cells were lysed and the lysate added to new macrophages. Two hours post infection the cells were lysed and intracellular bacterial counts determined. **G**) shows a schematic illustration of this reinfection as well as data on intracellular bacteria 2h post infection as determined by plating of cell lysates.

The gene expression data, together with the fact that TCSs are commonly involved in bacterial adaptation to environmental stimuli, suggests a role for Ihk/Irr in the early adaptive phase of *S. pyogenes* to the macrophage intracellular environment. Our recent study [6] demonstrated that at later time-points, following a replicative phase, *S. pyogenes* egress out of the cells, at which point they are fit to infect new cells. Thus at this point, the bacteria must be equipped with appropriate virulence factors for infectivity. It seems unlikely that the Ihk/Irr would also support the later phases of infections considering that only few virulence genes has been reported to be regulated by Ihk/Irr. To test this, the gene expression experiments were extended to include a 6h time point, representing a late phase of macrophage intracellular infection. In addition to *ihk/irr*, the *emm*1 gene and the TCS CovR/S were selected for the analyses. The latter is a negative response regulator of many streptococcal factors including the capsule, which confers anti-phagocytic activity. Real-time qRT-PCR analysis revealed a pronounced shift between the two TCSs over time (Fig. 3A and B). During the early adaptation phase, both *ihk* and *irr* genes are highly up-regulated but their expression decreases as the infection proceeds. In contrast, *cov*R and *cov*S genes were markedly up-regulated at 6h when compared to 2h pi. Interestingly, the highest expression of *ihk/irr* was seen at 2h pi, whereas at 1h pi the levels were lower, although not as low as at 6h pi (3D). As we are using an infection model without centrifugation, bacterial uptake occurs over an extended period, which results in a heterogeneous intracellular bacterial population with respect to the stage/phase of infection. Thus, samples at 1h pi are likely to include a substantial portion of bacteria that have just entered and not yet adapted to the intracellular environment. This in turn could explain differences in gene expression between 1h and 2h pi. In line with this reasoning, expression of *cov*R/S varied greatly between donors (3E). This temporal transition from Ihk/Irr to CovR/S is consistent with our hypothesis that the gene expression reverts from an adaptive/replicative profile to a profile that can promote bacteria-mediated cell lysis, infectivity and further dissemination of infection. To further support this, we analyzed the expression of the *has*A gene encoding for the anti-phagocytic hyaluronic acid capsule, which is one of the major genes suppressed by the CovR/S TCS [26]. qRT-PCR analyses showed that in 2 out of 3 experiments performed, the *has*A gene was 8- to 4-fold down-regulated at 6h pi, whereas in one experiment it remained unaltered. Although not under the control of either Ihk/Irr or CovR/S, another gene of interest is the *emm*1 gene, due to its previously shown importance for intracellular survival [6]. Our microarray data from the 2h infected cultures did not reveal a deregulated expression. However, qRT-PCR analyses showed in average a 2-fold increase in expression of the *emm*1 gene in bacteria recovered 6h pi when compared to 2h pi (p  =  0.03)(Fig. 3C). Interestingly, the lowest expression of *emm*1 was seen at the 1h time point (Fig 3F).

To functionally assess whether these differences in gene expression between 2h and 6h makes the strains more fit to egress and infect new cells, a re-infection assay was established in which bacteria were collected from cells infected for 2h and 6h pi. The recovered bacteria were then allowed to re-infect new cells for 2h after which intracellular bacterial load was determined. In line with our hypothesis, data from two separate experiments revealed markedly higher bacterial load in cells infected with bacteria recovered from 6h pi when compared to bacteria from 2h pi cultures (Fig. 3G).

### Importance of Ihk/Irr in Intracellular *S. pyogenes*

**Figure 4:**
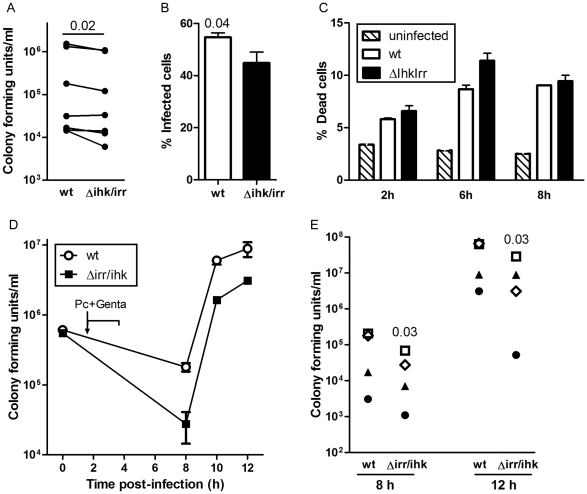
Reduced bacterial counts of an ihk/irr-deficient mutant, as compared to wildtype **S. pyogenes.** Monocyte-derived macrophages were infected for various time points with either 5448 WT strain or an isogenic deletion mutant (Δihk/irr). The cells were infected with equal infectious doses and the infection was allowed to proceed for 1-2h depending on assay after which extracellular bacteria were eradicated by antibiotics. **A**) Colony forming units plated from macrophage lysates at 2h pi according to material and methods. Data from 7 donors, **B**) shows the mean % ± SEM of cells harboring viable cocci as determined by bacterial viability stain at 4 h pi (n = 7 donors), **C**) Percent dead cell marker positive cells at various time points after infection as analyzed by flow cytometry (n = 2 donors). The bars show mean ± SEM of duplicate samples from one representative donor. **D**) At 4h pi, antibiotic-free media was added and bacterial load as monitored by plating cell culture media at defined time points shows the results of infection of cells from one representative donor, and (**E**) compiled data from 5 experiments using cells from different donors (indicated by separate symbols). Note that due to the log scale, some symbols overlap. Statistically significant differences were determined by use of paired Wilcoxon signed rank test and *p* values are indicated in the graphs.

To further test whether Ihk/Irr contribute to intracellular survival of *S. pyogenes* in human macrophages, an isogenic *ihk/irr*-deficient mutant (Δihk/irr 5448) was generated and its intracellular survival was compared to that of the wild-type (WT) parent strain (Fig. 4). Infection frequency was assessed by lysis of infected cells and enumeration of colony forming units (CFU), as well as by microscopic assessment of the percentage of cells harboring viable bacteria. The results revealed that infection with the Δihk/irr 5448 was associated with somewhat lower intracellular bacterial counts (Fig 4A), as well as lower % of infected cells at 4h pi, when compared to the wt 5448 (Fig 4B). Two donors were monitored over time (4–8h) for % infected cells and corroborating our previous data with respect to wt 5448 [5], the frequency of infected cells decreases over time, while no differences between Δihk/irr and wt 5448 infected cells could be observed (data not shown). Cell viability was assessed by flow cytometry through staining with a membrane impermeable dead cell marker. Our results reveal that *S. pyogenes* infection results in 6–12% cell death, as compared to 2–4% for uninfected cells (Fig 4C). Importantly, there were no differences in cellular death between cells infected with either strain (Fig. 4C). However, monitoring the infection and egress of bacteria over time by determining bacterial CFUs in antibiotic-free cell cultures revealed consistently lower bacterial counts in cultures infected with the mutant when compared to the WT (*P*  =  0.03) (Fig. 4D and E). Taken together, our data confirmed a positive effect of the Ihk/Irr TCS on bacterial load in cultures of infected macrophages, due to either enhanced intracellular survival and/or enhanced egress of the bacteria out of the cells.

## Discussion

*S. pyogenes* is a versatile and efficient colonizer of many environments by virtue of its sophisticated ability to adapt to the changing milieu both with respect to metabolic processes and virulence profile [9], [27]. Here we focus on transition of *S. pyogenes* from an extracellular to an intracellular pathogen in human macrophages and we demonstrate that 145 genes are differentially transcribed during the early adaptation phase. Many of the up-regulated genes have established functions in cell wall synthesis and metabolism. As such they include several ABC transporters, which can contribute to bacterial metabolism and virulence through import/export of ions and nutrients [28]. Among the transporter genes, the ABC transporter system Spy2032/2033 demonstrated the highest up-regulation (8- and 14-fold in microarray and qRT-PCR analyses, respectively) in intracellular when compared to extracellular bacteria. Interestingly, these genes were also found to be up-regulated during infection in human whole blood [29] and thus seem to be important during *S. pyogenes* infections in humans.

Considering that many important virulence factors are associated with mobile genetic elements [30], it was noteworthy that many of the phage-encoded genes were up-regulated in intracellular bacteria. Most of these deregulated phage-encoded genes encoded for hypothetical proteins with unknown functions, and none of the classical phage-encoded virulence factors, such as streptococcal pyrogenic exotoxin (Spe) A, SpeC, and DNases (Streptodornase and mitogenic factors), were identified. The importance of prophages in the evolution of diversification and emergence of hyper-virulent streptococcal clones is well recognized [31], [32]. Aziz et al [32] compared the hyper-virulent M1T1 5448 strain (i.e. same strain as in present study) with the laboratory reference M1T1 strain SF370. The analyses revealed that the two strains share a 95% homology, and notably the majority of the differential 5% consists of phage related sequences being present in the hyper-virulent clinical M1T1 isolate [32].

A major finding of our microarray analysis was the up-regulation of genes encoding the TCS Ihk/Irr during the early phase (i.e. 2h) of infection, indicating that this TCS influences gene expression supporting intracellular bacterial persistence in macrophages. The Ihk/Irr system was first discovered by Federle et al [12] as a TCS with homology to the PhoPS response regulator in *Bacillus subtilis* [33]. The PhoPS system is widely distributed among bacterial species and is involved in regulating virulence factors through sensing of the phosphate concentration in the surrounding environment, including intracellular survival of *Salmonella ssp., Enterococcus faecalis* and *Listeria monocytogenes* in macrophages [34]–[39]. qRT-PCR analyses demonstrated a temporal expression of TCSs, evident by a down-regulation of the *ihk/irr* genes as the infection proceeds, while the TCS *cov*R*/cov*S genes are up-regulated at 6h compared to 2h pi. In contrast, gene expression of *ihk/irr* at 1h pi was lower than at 2h pi. This is likely a consequence of the fact that our infection model does not employ centrifugation but allows a more physiologic prolonged bacterial uptake process. As a result, samples from the 1h time point represent a diverse bacterial population as the cells are likely to contain bacteria that have been intracellular for various periods of time ranging from 10 minutes to 1h. Taken together, our findings suggest that there is a fine-tuned gene regulation during the different phases of infection with activation of Ihk/Irr during the early adaptation phase. At later phases of infection the bacteria egress out of the cells and prepare to infect new cells. This in turn requires another gene expression profile, potentially influenced by CovR/S. Regulation of gene expression by CovR/S is complex. Whereas it is considered a negative response regulator that turns off expression of virulence genes, certain mutations in CovS have been associated with up-regulation of certain CovR regulated genes. A major target of the CovR repressor is the anti-phagocytic hyaluronic acid capsule. Acapsular variants have previously been shown to be more efficient in adherence to and entry into cells [40], [41]. In line with this reasoning, analyses of expression of the *has*A gene indicated a down-regulation at the 6h time point. Also the M-protein, which is not directly regulated by CovR/S but has been strongly implicated as a key factor for survival in phagocytic cells, showed an upregulated gene expression at 6h pi. To test whether these noted differences in gene expression translate into differential infectivity, bacteria recovered at early (2h) and later (6h) stages of infection were used in a re-infection assay. Indeed, bacteria from the 6h cultures (associated with low capsule and higher *emm*1 expression) were found to be more infectious than those recovered during early stages (2h pi).

Further support for a role of Ihk/Irr for intracellular *S. pyogenes* in macrophages was sought by generating an isogenic *ihk/irr* deficient mutant and comparing its survival to that of the wt strain. A slight but significant reduction in intracellular bacterial counts, % infected cells, as well as lower bacterial counts recovered in antibiotic-free media after infection were observed following infection with the mutant when compared to the wt strain. Ihk/Irr has previously been reported to be involved in *S. pyogenes* resistance to phagocytic killing in human neutrophils by conferring resistance to reactive oxygen species and antimicrobial peptides [22], [25]. Other investigations involving *in vivo* studies of *S. pyogenes* in a mouse model [42] and an *in vitro* system with human whole blood [29] or saliva (14) have reported an activation of this system during infection. In contrast to the noted association between Ihk/Irr and resistance to reactive oxygen species in neutrophils [22], we found that oxidative stress response genes were down-regulated at 2h pi in macrophages. One plausible explanation to our results is that *S. pyogenes* survival in macrophages is critically associated with an arrest in phagolysosomal maturation [6], which likely limits exposure to reactive oxygen species and thereby, the induction of oxidative stress responses. Another contributing factor could be differences between serotypes, as the functional studies in neutrophils were done using an M6 *S. pyogenes* strain in contrast to M1 used in this study. Thus, the exact function of Ihk/Irr intracellularly and in different serotypes remains to be defined in future studies.

## Materials and Methods

### Ethics Statement

This study includes blood samples from healthy volunteers or buffy coats of blood provided by the blood bank at the Karolinska University Hospital. The buffy coats were provided anonymously; hence informed consent was not required. In case of healthy volunteers, donors were individuals well acquainted with the research conducted; thus, verbal informed consent was deemed sufficient and was documented in laboratory journals. The ethical research committee at Huddinge University Hospital (Forskningskommitté Syd) approved the study including this consent procedure.

### Primary Human Cell Preparation and Culture Conditions

Human monocyte-derived macrophages were prepared from healthy blood donors essentially as described [6]. Briefly, monocytes were isolated from buffy coats using RosetteSep (Stemcell Technologies) separation followed by a Lymphoprep (Axis-Shield) centrifugation step. Monocytes were then seeded in 6-well low-adherence cell culture plates (Corning) in RPMI containing 10% FCS and 50 ng/ml macrophage colony-stimulating factor (Immunotools), and cultured for 5–7 days to obtain macrophages.

### Bacterial Strains

A clinical M1T1 isolate of *S. pyogenes* (strain 5448) (36) and the isogenic mutant 5448Δ*ihk-irr* were grown at 37°C to stationary phase in Todd-Hewitt broth supplemented with 1.5% yeast extract and diluted to the required inoculum depending on experimental assay. The two strains had equal growth rates.

### Generation of *ihk-irr* Allelic Replacement Mutant

The isogenic mutant 5448Δ*ihk-irr* was constructed by an allelic exchange mutagenesis procedure developed for *S. pyogenes* [43]. Briefly, DNA sequences encoding ∼1 kb upstream and ∼1 kb downstream of the target genes were amplified from the M1T1 GAS 5448 chromosome using *PfuUltra*® II (Stratagene) and the following primer pairs: (a) Ihk-Irr-UpFwd (5′-Cgactcgaggaagtgatgggaaatcccttcaggg-3′) + Ihk-Irr- UpRev (5′-cca gtgatttttttctccaTaacttccctttctagattggcg -3′ with 20 bp 5′ extension matching the 5′ end of *cat* gene); (b) Ihk-Irr-DownF (5′-tggcagggcggggcgtaatccatactagtaggacgacaaaagtc-3′ with 19 bp 5′ extension matching 3′ end of *cat* gene) + Ihk-Irr-DownR (5′-cgagaattccccataacggcgtgacaacaggttg-3′). The chloramphenicol acetyltransferase gene (*cat*) was amplified from plasmid pACYC184 using primers Cat-For (5′-atggagaaaaaaatcactggatatacc-3′) and Cat-Rev (5′-ttacgccccgccctgccactcatcgca-3′). Fusion PCR was performed with the Failsafe enzyme (Epicenter), the Ihk-Irr-Up-Fwd and Ihk-Irr-Down-Rev primers, and the three above-mentioned amplicons, to generate a PCR product in which *cat* precisely replaced Ihk-Irr in the GAS chromosomal context. The fusion construct was subcloned into temperature-sensitive plasmid pHY304 that carries an erythromycin-resistance (Em^R^) marker. The knockout plasmid was transformed into 5448 WT with selection for Em^R^ (2μg/ml) at the permissive temperature (30°C), then single-crossover events identified after shifting the culture to the non-permissive temperature (37°C) maintaining Em selection. The culture was relaxed by serial passage at 30°C without antibiotics, and the double-crossover event identified by screening for colonies with a Cm^R +^ Em^S^ phenotype at 37°C. The precise in-frame allelic replacement of *ihk-irr* with *cat* in the *S. pyogenes* 5448 chromosome was confirmed by PCR and sequence analyses.

### Infection Assay

Briefly, *S. pyogenes* were co-cultured with macrophages at 37°C and 5% CO_2_ for different time points. A multiplicity of infection (MOI) of 5–20 CFU/cell was used depending on the assay. At 1 h after infection, extracellular and adherent bacteria were killed by addition of 125 μg/ml gentamicin and 2.5 μg/ml penicillin G for 1 h, followed by washing in PBS twice, and addition of RPMI 1640 supplemented with 1 μg/ml penicillin G throughout the rest of the experiment.

### Expression Microarray Analysis

For comprehensive transcriptome analysis, oligonucleotides were spotted on glass microarrays (Microarrays, Inc.), representing all 2,346 open reading frames of the M1T1, strain SF370 of *S. pyogenes* [24] (GenBank accession #NC_002737), using oligo sets designed and provided by Drs. J. Scott and K. McIver, with additional open reading frames (mainly phage encoded) from strains MGAS8232 (GenBank accession# NC_003485) and MGAS315 (GenBank accession # NC_004070) [13], [14]. Each open reading frame was spotted in triplicates. RNA was extracted from intracellular bacteria (2h pi) using the Ribopure Bacteria kit (Ambion). Extracellular control bacteria were grown under identical settings, i.e. 6 well low-adherence plates, 37^o^C, 5% CO_2_, in cell culture media lacking cells and RNA was isolated as described above. Purified RNA was reversely transcribed into complementary DNA using SuperScript III reverse transcriptase (Invitrogen) and labeled with Alexa Fluor 546/647 by using a 3DNA Array 900 MPX kit (Genisphere). RNA was isolated from 3 biological replicas. The microarrays were scanned using a GenePix 4000B scanner and primary analyses were performed using GenePixPro software (version 4.0; Axon Instruments). The analyses included spot finding, alignment and adjustment, fluorescence normalization, flagging of poorly hybridized spots and background subtraction. Subsequent analyses were done using GeneSpring GX software (version7.3; Agilent technologies). To compare gene expression profiles of the two groups, GeneSpring’s parametric test was used and unequal variance was assumed. Microarray data validation was conducted using qRT-PCR. All raw microarray data was submitted to NCBI Gene Expression Omnibus (GEO) in accordance with MIAME standards (GEO accession numbers: GEO platform GPL14578 and samples GSM796440–41).

### Reverse-transcription (RT)-PCR

Real-time qRT-PCR analyses were conducted to validate microarray data and to obtain data on gene expression at 1h, 2h and 6h pi. RNA from intracellular and extracellular bacteria was extracted as described above, including a DNase step to secure removement of contaminating genomic DNA. RNA was then reversely transcribed using Quantitech reverse transcription kit (Qiagen) including control reactions without the RT enzyme to control for gDNA traces. Primers and TaqMan probes were designed using the PrimerExpress software (Applied Biosystems), shown in table S1. qPCR reaction was performed in the 7500 Fast system using 2x Gene Expression Master Mix (both from Applied Biosystems) and in accordance with provided protocols. Control experiments were performed with host cell cDNA to ensure no cross-reactivity of contaminating eukaryotic RNA with the prokaryotic target genes. The experiment was done with 3–6 biological replicates. Results from well triplicates were pooled and analyzed using the ΔΔCt method with extracellular *S. pyogenes* samples as calibrator and gyrase as endogenous control in MS Excel.

### Re-infection Assay

1×10^6^ macrophages were seeded/well in 6-well plates and infected for 1 hour with a MOI of 20 CFU/cell followed by antibiotic killing of extracellular bacteria for 1 hour as described above. After 2 hours and 6 hours, cells were washed twice with PBS where after cells were lysed with ddH_2_O (pH 11). The lysate was added to 5×10^4^ macrophages in a 48 well plate (duplicates). The lysate was also plated on blood agar plates to confirm an equal MOI between the two strains. After 2 hours of infection cells were lysed as previously described and bacterial content was quantified by plating lysates on blood agar plates.

### Lysis Assay

1×10^5^ macrophages were seeded in 24-well plates and infected for 1 hours with a MOI of 4–8 CFU/cell followed by antibiotic killing of extracellular bacteria for 1 hour. Cells were then washed 3 three times with PBS and lysed with ddH_2_O (pH 11). The lysate was plated on blood agar plates and bacterial content was quantified by counting colony-forming units.

### Quantification of Infected Cells with LIVE/DEAD Bacterial Viability Kit

1×10^5^ macrophages were seeded/well on cover slips in a 24-well plate. Cells were infected with *S. pyogenes* with a MOI of 10 CFU/cell for 2 hours after which antibiotics was added as described above. To detect intracellular bacteria, cells were incubated with LIVE/DEAD Backlight Bacterial viability kit (Invitrogen, Molecular Probes), which enables discrimination between viable and dead bacteria, in accordance with provided protocol. The number of cells harbouring > 2 viable bacteria was enumerated by microscopy analysis.

### Flow Cytometry

*S. pyogenes* were co-cultured with macrophages at 37°C and 5% CO_2_ for different time points. A multiplicity of infection (MOI) of 10 CFU/cell was used. At 1h post infection, extracellular and adherent bacteria were killed by addition of 1 µg/ml penicillin G into the supernating medium. At 2h, 6h and 8h post-infection, macrophages were harvested and stained with LIVE/DEAD Fixable Near-IR Dead Cell Stain (Molecular Probes) according to the manufacturer’s specifications. A positive control was included consisting of cells treated with 50% DMSO. 1×10^5^ cells were acquired using an LSRFortessa (BD) cell analyzer and analyzed using FlowJo (Tree Star) software.

### Exit-assay

1×10^5^ macrophages were seeded/well in a 24-well plate. Cells were infected with a MOI of 5 CFU/cell for 2 hours. Extracellular bacteria were eradicated by adding antibiotics as described for the infection assay above. After 4 hours of infection, cells were washed three times to remove antibiotics and antibiotic-free media was added. Cells were further incubated and at specific time points the amount of egressed bacteria was enumerated by plating samples of media.

### Statistical Evaluation

Data were analyzed by GraphPad Prism version 4.0 for Windows (GraphPad software, San Diego, CA). Statistical significant differences between groups were determined by paired Wilcoxon signed rank test.

## Supporting Information

Table S1 Sequences of primers and TaqMan probes designed and used in this study.
(TIF)
