# Supplementary figures and images for: Intracellular Streptococcus pyogenes in Human Macrophages Display an Altered Gene Expression Profile
Source: PLoS One. 2012 Apr 12;7(4):e35218. doi: 10.1371/journal.pone.0035218 (PMC3325220; doi:10.1371/journal.pone.0035218)

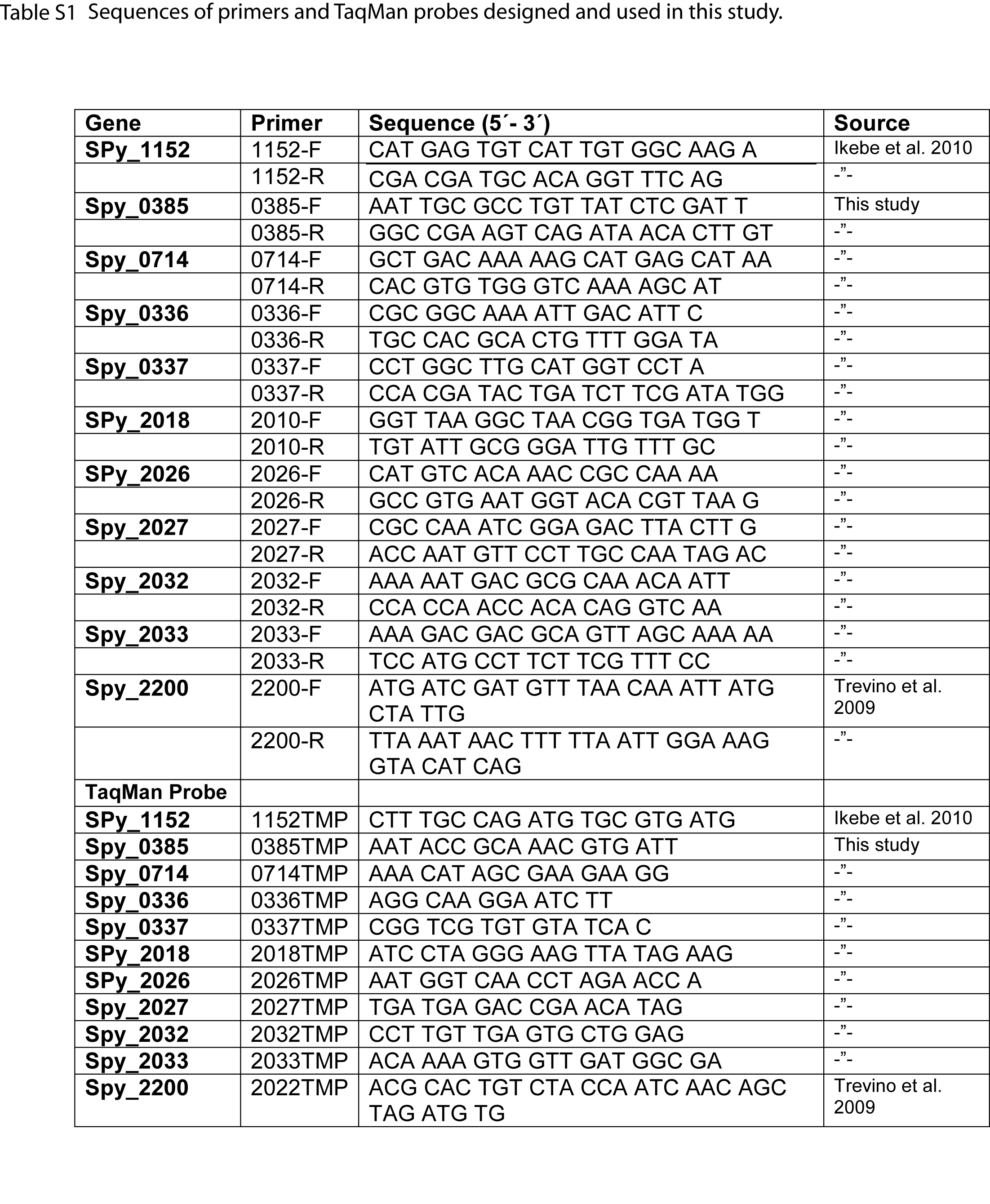

Supplement: Table S1 — Sequences of primers and TaqMan probes designed and used in this study. (TIF) [file pone.0035218.s001.tif]
